# A Long-Term Comparison of Periprosthetic Femoral Bone Mineral Density Between a Short and a Standard Stem Used in Total Hip Arthroplasty

**DOI:** 10.7759/cureus.110276

**Published:** 2026-06-04

**Authors:** Solène Maziere, Shirin Monadjemi, Roger Erivan, Stéphane Boisgard, Samuel Adelou, Sandrine Malochet-Guinamand, Guillaume Villatte, Stephane Descamps

**Affiliations:** 1 Orthopedic Surgery, Centre Hospitalier Universitaire de Clermont-Ferrand, Clermont-Ferrand, FRA; 2 Direction de la Recherche Clinique et de l’innovation (DRCI) (Clinical Research and Innovation Department), Centre Hospitalier Universitaire de Clermont-Ferrand, Clermont-Ferrand, FRA; 3 Rheumatology, Centre Hospitalier Universitaire de Clermont-Ferrand, Clermont-Ferrand, FRA

**Keywords:** bone mineral density, short stems, standard stem, stress shielding, total hip arthroplasty

## Abstract

Introduction and aim: Short femoral stems in total hip arthroplasty (THA) were developed to preserve proximal femoral bone stock by minimizing stress shielding. However, their long-term outcomes remain debated when compared to standard-length stems. We hypothesized that short stems would allow better preservation of proximal periprosthetic bone mineral density (BMD) compared to standard stems. The primary objective of this study was to evaluate long-term periprosthetic BMD in patients receiving either short or standard femoral stems, with the hypothesis that short stems would provide superior preservation of proximal bone. The secondary objective included the comparison of clinical and radiological outcomes.

Methods: This single-center retrospective study included patients who underwent THA between 2010 and 2018 using either the Optimys short femoral stem (Bettlach, Switzerland: Mathys) or the standard-length twinSys stem (Bettlach, Switzerland: Mathys). Periprosthetic BMD was evaluated using dual-energy X-ray absorptiometry (DEXA) across six modified Gruen zones. Radiographs were evaluated by two reviewers and clinical outcomes were evaluated using the Harris Hip Score (HHS) and the presence or absence of thigh pain.

Results: At a mean follow-up of 9.4 years, the analysis of 24 hips (10 short stems, 14 standard stems) revealed no significant differences in periprosthetic BMD. Clinical outcomes, including HHS (median {IQR}; standard: 95.8 {80.8-100.0}; short: 88.4 {80.0-93.0}) and thigh pain incidence, were comparable, and radiographic evaluation revealed no difference in stress shielding or osteolysis.

Conclusions: Our findings suggest that, over the long term, both the Optimys and twinSys stems are subject to stress shielding. These results contrast with some prior studies and highlight the potential influence of stem design and follow-up duration on bone remodeling outcomes.

## Introduction

Aseptic loosening remains one of the most common complications following total hip arthroplasty (THA) [1]. Among its multifactorial etiologies, stress shielding plays a significant role by inducing periprosthetic bone remodeling [2,3]. This phenomenon is especially relevant when using conventional uncemented femoral stems. The stiffness, geometry, and fixation pattern of the femoral component modify proximal femoral load transfer, potentially leading to stress shielding and periprosthetic bone remodeling [4-6]. Typically, the load is transferred distally to the diaphyseal cortex, resulting in cortical hypertrophy, whereas the offloaded metaphysis undergoes bone resorption [6,7]. This proximal bone loss reduces the bone-implant interface, potentially increasing the risk of periprosthetic fractures [5,8].

To address these issues, shorter stems were developed. These are designed to promote more physiological load transfer while maintaining axial and rotational stability. The design of short stems we study here (Optimys) not only helps minimize stress shielding but also requires less femoral neck resection and diaphyseal reaming, thereby preserving proximal bone stock for future revisions, making them particularly advantageous in young and active patients [5,9]. Additionally, they require a smaller incision during surgery, resulting in less soft-tissue disruption and reduced thigh pain [9,10].

Although there is currently no universally accepted definition, short-stem implants are generally characterized by a length of less than 120 mm and by achieving primary fixation within the metaphyseal region [5,11]. Several classification systems, including the French Hip and Knee Society (SFHG) [10], have been proposed to categorize the wide variety of short stems now available on the market [10,12,13]. The growing variety of these stems has also led to a surge in related clinical publications. Most have demonstrated favorable outcomes [3,14-19], including superior bone preservation with bone mineral density (BMD) measurements, although others have reported mixed results [20-22]. However, evidence regarding long-term outcomes remains limited [3,9].

## Materials and methods

Study design

**Figure 1:**
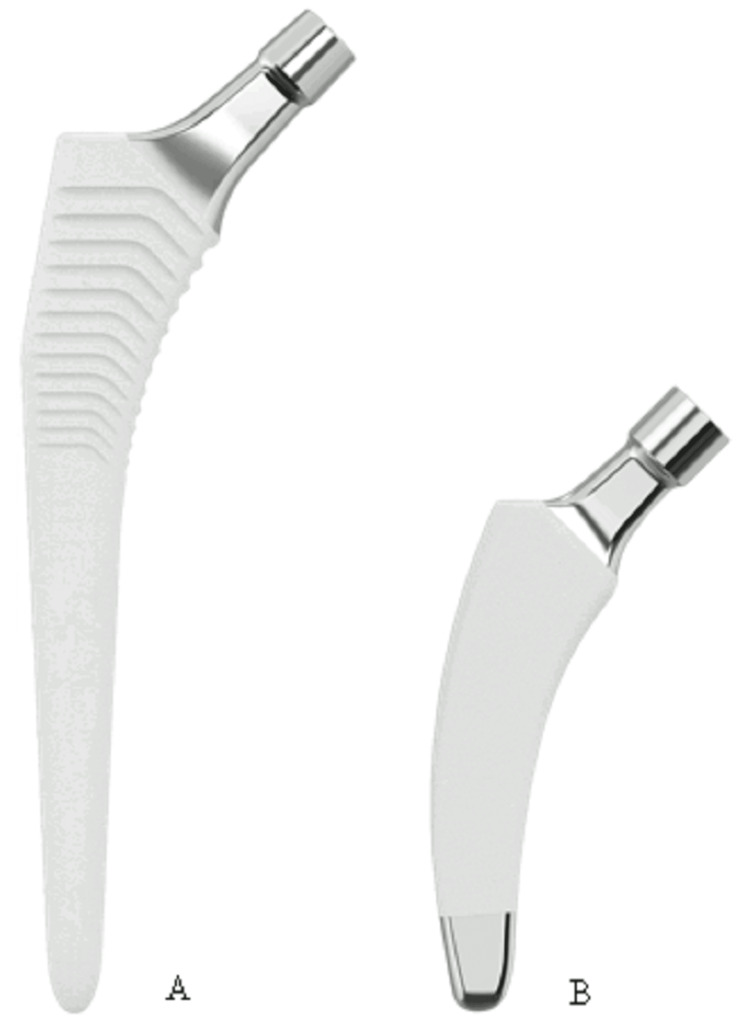
The two stem designs used in the present study. (A) TwinSys standard stem and (B) Optimys short stem.

This single-center, retrospective, comparative study was approved by our institutional review board. Eligible patients were identified retrospectively and evaluated prospectively at consultation visits occurring at least five years post-THA. The first type of stem selected as a short stem was the uncemented Optimys femoral stem (Bettlach, Switzerland: Mathys), a commonly used implant in our institution, and classified as a type-3 stem according to the French Hip and Knee Society (SFHG) classification (Figures 1A, 1B) [10]. An initial design involving patients with bilateral THA, with one hip implanted with the Optimys stem and the other with a standard stem, was planned to reduce interindividual variability and compensate for the lack of preoperative BMD data. However, this was abandoned due to an insufficient number of eligible cases. The final study comprised two groups. The study included patients in the first group who received a unilateral uncemented Optimys short stem (fixed neck, anatomical curvature that helps restore the patient’s center of rotation and femoral offset, and hydroxyapatite coating, except for its polished distal tip) between 2013 and 2018. The control group consisted of patients who were implanted with an uncemented twinSys standard femoral stem (tri-tapered, with a pronounced proximal rib structure; Bettlach, Switzerland: Mathys) prior to 2018 (Figures 1A, 1B). We included patients aged ≥18 years who had undergone THA for primary coxarthritis. Exclusion criteria were as follows: THA indications other than coxarthritis, prior surgery on the index hips, metabolic bone disease, postoperative complications, cognitive impairment, residence more than 60 km from the center, or medical conditions precluding attendance at follow-up visits. Written informed consent was obtained from all participants.

**Figure 2:**
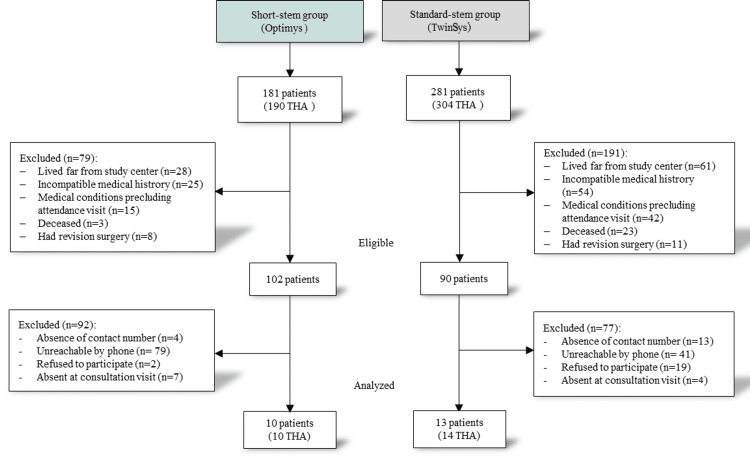
Flow diagram summarizing the study design. THA: total hip arthroplasty

In the short-stem group, 181 patients were identified. Of these, 79 did not meet the inclusion criteria, and an additional 92 were excluded due to loss to follow-up, refusal to participate, or inability to contact, leaving 10 patients for analysis. In the standard-stem group, 281 patients were screened; 191 did not meet the inclusion criteria, and 77 were excluded for similar logistical reasons, leaving 13 patients for final analysis. The patient selection process is summarized in the flowchart (Figure 2).

Materials

All patients were operated on using the anterolateral approach with previously described stems. They were operated on by five different specialist surgeons with an experience of five to 30 years. Acetabular components included either a single-mobility monoblock cup (RM Pressfit vitamys; Bettlach, Switzerland: Mathys) or a dual-mobility cup (Novae Sunfit; Décines, France: Serf). Femoral heads were metallic or ceramic, depending on patient characteristics and surgical indication. All patients followed the same postoperative protocol, allowing immediate full weight-bearing.

Assessment methods

**Figure 3:**
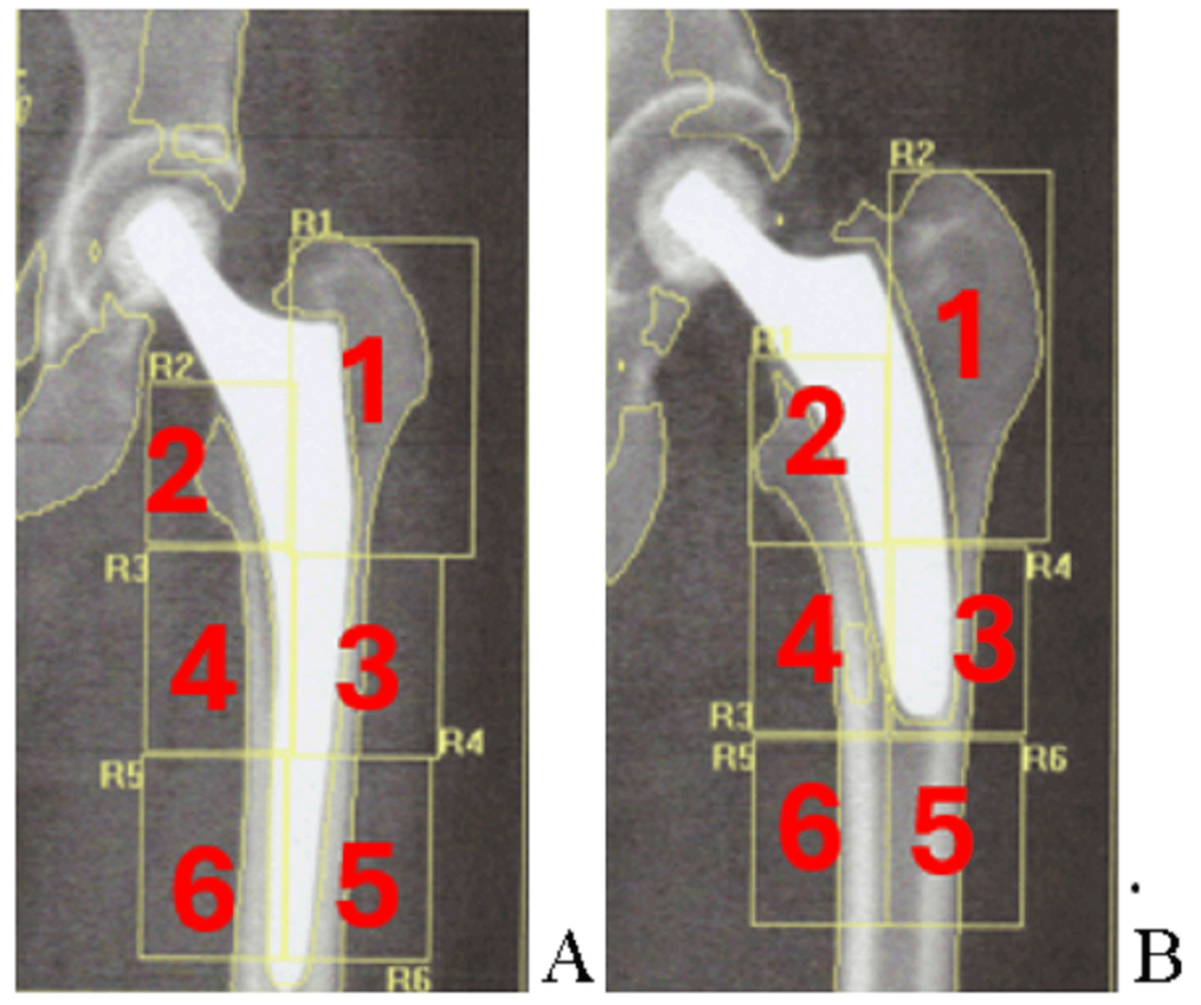
Adaptation of the Gruen classification to compare the six zones in stems of different lengths. (A) TwinSys standard stem and (B) Optimys short stem.

Patient medical records were reviewed to collect demographic data, acetabular cup type, and femoral bone quality. The latter was assessed according to Dorr’s classification [23]. The canal flare index (CFI) was defined as the ratio of the medullary canal width 2 cm above the intertrochanteric line to the femoral canal width at the isthmus in the anteroposterior view. Consultation visits were scheduled at a minimum of five years post-THA, during which primary and secondary endpoints were evaluated. The primary endpoint was the difference in periprosthetic BMD between the two groups. Secondary endpoints included clinical, functional, and radiological outcomes. Periprosthetic BMD was measured using dual-energy X-ray absorptiometry (DEXA) with the Discovery QDR A system (Bedford, MA: Hologic, Inc.). Scans were performed with patients in the supine position and the affected hip in 15° internal rotation. BMD was analyzed using dedicated software (Hologic Discovery A version 13.3.0.1) in a metal-removal mode to enhance contrast and improve accuracy. Radiological evaluation included anteroposterior pelvic radiographs, with the most recent images assessed for signs of stress shielding according to Engh’s classification by two observers, according to the result after discussion [6]. Gruen zones were modified to align with femoral anatomy, enabling consistent and symmetrical analysis of the bone-prosthesis interface across both stem types. Following a published method, six regions of interest (ROI) were defined [20]. ROI-1 (size: 84×28 mm) was centered on the greater trochanter, and other ROIs were equal-sized (41×28 mm), with one on the lesser trochanter and two lateral and two medial (Figures 3A, 3B). The mean BMD (g/cm²) for each ROI and the overall BMD (global mean BMD) were calculated for each group. Functional outcomes included the Harris Hip Score (HHS), and the incidence of thigh pain was also recorded by a simple yes/no question.

Statistical analysis

Statistical analyses were performed using Stata software version 19 (College Station, TX: StataCorp LLC), with a two-tailed alpha level of 5%. To prevent confounding bias and address data dependency issues, the analysis was conducted using only one prosthesis per patient. For the patient with a hybrid bilateral implantation, only the Optimys stem was included; for the bilateral twinSys case, only one of the two stems was selected. Quantitative variables were presented as medians with interquartile ranges (IQR) and compared between the two groups (Optimys vs. twinSys) using the Mann-Whitney U test. Qualitative variables were expressed as frequencies and percentages, then compared using Fisher's exact test. Finally, a sensitivity analysis was performed using the hip as the statistical unit. These univariate analyses were supplemented by mixed-effects models for the primary endpoints to account for intrapatient correlation in cases of bilateral surgery.

## Results

Demographic and baseline characteristics of both study groups are summarized in Table 1. The short-stem group included 10 patients, while the standard-stem group included 13 patients. One patient had a bilateral twinSys implant, while two patients received both an Optimys and a twinSys stem. For these two bilaterally operated patients, only the Optimys stem was included in the analysis, resulting in 11 patients in the standard-stem group. No statistically significant differences were identified between the groups across baseline variables. The mean follow-up duration for all patients was 9.4±3.2 years, with a median of 9.8 years (IQR: 7.5-9.9) in the short-stem group and 11.5 years (IQR: 6.7-13.7) in the standard-stem group. Age at surgery was similar between groups, with a median age of 60.0 years (IQR: 55-68) in the short-stem group and 60.0 years (IQR: 57-65) in the standard-stem group. A higher proportion of female patients was noted in the short-stem group (70%; n=7) compared to the standard-stem group (27%; n=3), although this difference was not statistically significant. Preoperative canal flare index (CFI) values were comparable in both groups (3.2 for the short stem vs. 3.4 for the standard stem). Regarding proximal femoral morphology, Dorr type A, characterized by thick cortices and a narrow canal, was observed in one hip (10%) in the short-stem group and two hips (18%) in the standard-stem group. Most patients in both groups exhibited Dorr type B morphology, with nine hips (90%) in the short-stem group and nine hips (82%) in the standard-stem group (p=1). No cases of Dorr type C femoral morphology were identified. Mean postoperative periprosthetic BMD values by regions of interest (ROI) are presented in Table 2. No statistically significant differences in BMD were identified between the two cohorts. In both cohorts, region-1 consistently showed the lowest BMD, followed by region-2. Postoperative clinical and radiological results revealed no intergroup differences in terms of thigh pain or evidence of stress shielding (Table 2). At the last follow-up, the median HHS was higher in the twinSys group compared to the Optimys group (97.8 vs. 88.4), although this difference did not reach statistical significance.

**Table 1:** Demographic data of patients. *Number of hips. **P-value <0.05 is considered significant. CFI: canal flare index

Variables	Standard stem, twinSys (11 patients, 11 hips)	Short stem, Optimys (10 patients, 10 hips)	p-Value**
Age (years), median (IQR)	60.0 (57-65)	60.0 (55-68)	0.89
Gender, n (%)			
Male	8 (73)	3 (30)	0.09
Female	3 (27)	7 (70)	
Body mass index (kg/m²), median (IQR)	28.3 (25.2-31.0)	29.5 (23.5-32.0)	0.89
Operated side, n* (%)			
Right	7 (64)	3 (30)	0.20
Left	4 (36)	7 (70)	
Follow-up duration (years), median (IQR)	11.5 (6.7-13.7)	9.8 (7.5-9.9)	0.16
Noble-CFI, median (IQR)	3.4 (3.0-4.5)	3.2 (3.1-4.3)	0.77
Dorr femoral bone geometry, n* (%)			
A	2 (18)	1 (10)	1
B	9 (82)	9 (90)	
C	0	0	

**Table 2:** Osteodensitometry results according to the adapted regions and long-term clinical and radiological results. *Number of hips. **One missing value in the Optimys group. ***P-value <0.05 is considered significant. Stress shielding grade according to Engh’s classification: 1 - absence or rounding off of the proximal medial neck; 2 - loss of the medial cortex density around the minor trochanter; 3 - loss of the medial cortex density below the minor trochanter; and 4 - cortical resorption into the diaphysis. BMD: bone mineral density; HHS: Harris Hip Score

Variables	Standard stem, twinSys (11 hips)	Short stem, Optimys (10 hips)	p-Value***
BMD (g/cm²), median (IQR)			
Global	1.09 (0.98-1.26)	1.07 (1.04-1.18)	0.67
Region-1	0.71 (0.65-0.80)	0.66 (0.60-0.75)	0.29
Region-2	0.87 (0.71-0.97)	0.71 (0.67-0.83)	0.18
Region-3	1.52 (1.35-1.61)	1.59 (1.46-1.72)	0.44
Region-4	1.53 (1.33-1.73)	1.65 (1.43-1.82)	0.57
Region-5	1.60 (1.53-1.82)	1.56 (1.54-1.68)	0.36
Region-6	1.74 (1.61-1.92)	1.69 (1.62-1.88)	0.53
HHS	97.8 (80.8-100.0)	88.4 (80.0-93.0)	0.06
Presence of thigh pain, n* (%)	3 (27)	3 (30)	1
Stress shielding grade**, n (%)			
1	5 (50)	4 (44)	1
2	4 (40)	4 (44)	
3	1 (10)	1 (11)	
4	0	0	

## Discussion

As the number of THA procedures continues to grow, short-stemmed prostheses are increasingly recognized as a viable alternative to standard-length stems in THA, especially in younger patients for whom long-term bone preservation is crucial [24]. As their clinical use expands, it is important to understand the periprosthetic bone response following implantation to optimize outcomes and ensure implant longevity. The present study demonstrated that, overall and at a mean follow-up of 9.4 years, BMD in the short-stem group was comparable to that of the standard stem group. Notably, region-1 exhibited the greatest bone loss in both groups, suggesting that, despite the use of a shorter stem design, load transfer may still predominantly occur in the distal rather than the proximal femur.

Short stems are designed to promote proximal load transfer and minimize stress shielding in the metaphyseal region. However, stress distribution appears to depend more on stem geometry than on length. A finite element study of collared neck stems showed that stems with identical geometries but different lengths produced similar stress distribution patterns, while the presence of a collar significantly influenced stress concentration in the proximal region [25]. Regarding in vivo studies, numerous publications exist, but comparison remains challenging due to the differences in implant morphology, fixation sites, and the reference selected as a control (Table 3). It appears that the length is probably a little factor in all that can influence the stress distribution and bone density.

**Table 3:** Comparison of different studies on short-stem implants with bone mineral density (BMD) analysis. Number: short stem/conventional stem at last follow-up. Stem type was defined according to the SFHG classification [10]. SFHG: French Hip and Knee Society

Studies	Implant	Stem type	Follow-up (years)	N hips (short/standard group)	Comparison	Baseline reference	Significant changes in BMD at last follow-up
Hirao et al. [19]	Taperloc Microplasty	III	5	17/17	Standard stem in contralateral femur	Within 3 months postoperatively	↓ Zone 7 in both groups. Fewer BMD changes in zone 2 in the short-stem group
Freitag et al. [20]	Fitmore	III	1	51/81	Standard stem	1 week postoperatively	↓ Zone 2, 4, 6, 7 for both stems (more pronounced in zone 7), ↓ zone 1 for short stem, ↓ zones 3 and 5 in the conventional stem
Chen et al. [26]	Mayo	III	5.7	29/0	Contralateral femur	-	↑ Zone 3 (+20.9%) and 2 (+9.2%), ↓ zone 1 (-14.4%)
Samy and El-Tantawy [27]	MiniHip	III	1.8	25/25	Standard stem	1 week postoperatively	↓ Zone 1 in both groups, ↓ zone 7 in the standard group
Jahnke et al. [28]	Metha	III	1	40/0	-	1 week postoperatively	↓ Zone 1 (-8.0%) compared to 1 week postoperative, ↓ zone 7 (-11.4%) and zone 4
Zeh et al. [15]	Nanos	III	1	23/0	-	Immediate postoperatively	↑ Zone 6 (+12%), ↓ zone 1 (-15%), 2 (-5%), and 7 (-12%)
Salemyr et al. [16]	Proxima	IV	2	24/23	Standard stem	2 days postoperatively	↓ Zone 1 in both groups, standard group (-2.5%), short-stem group (-20.6%), ↓ zone 7 for both groups, ↓ globally for both groups (significantly lower bone resorption in the short-stem group)
Lerch et al. [14]	Metha	III	2	25/0	-	1 week postoperatively	↑ Zone 6 (+10.6%), ↓ zone 3 (-8.8%) and 1 (-7.7%)
Djebara et al. [29]	Optimys	III	4	47/0	Contralateral femur	1 year	↓ Zone 2 (-13.7%)
Djebara et al. [29]	Vitae	IV	4	46/0	Contralateral femur	1 year	↓ Zone 2 (-4.8%), ↓ zone 6 (-11%) and 7 (-5.8%)
Kim et al. [30]	Proxima	IV	4.5	144/0	-	1 week postoperatively	↑ Zone 1 (+3%), ↓ non-significant in zone 7
Koyano et al. [3]	CenTpillar	V	9.2	36/36	Standard stem in contralateral femur	-	Higher prevalence of cancellous condensation in L2, L3, M2, M3 on short stem, greater BMD in L3, M3, M4 in the short stem. Significantly lower in G2, G3, and G6 in the short stem. No perioperative data
Kim et al. [9]	Proxima	IV	17.5	858/858	Standard stem	1 week postoperatively	↓ Zones 1 and 7 in both groups. But significant only in the standard stem

As reported in several publications, it seems that stress shielding in specific regions, particularly ROI-1 and ROI-7, remains a challenge even for short stems [15,20,26,27]. For instance, Freitag et al. found significant BMD loss in the proximal femur in both Fitmore and standard stems [20]. Although BMD recovery was observed below the calcar between three months and one year with the Fitmore stem, stress shielding persisted at final follow-up. Similar to our findings, they concluded that neither implant design fully prevented bone loss. However, their follow-up was limited to one year. Other short-stem designs have shown comparable results. A study using Nanos stems reported a constant decrease in BMD in zones 1, 2, and 7, with bone loss in zones 1 and 7 reaching 15% and 12%, respectively [15]. Similarly, a publication based on unilateral Metha stems with a two-year follow-up observed that the greatest bone loss was at the greater trochanter [14], a finding consistent with a study using MiniHip stems, in which most bone loss occurred during the first six postoperative months [27].

Some studies indicate that the most significant changes in periprosthetic BMD occur within the first postoperative year and tend to stabilize thereafter. However, early postoperative BMD measurements may be confounded by temporary factors such as surgical disruption of blood supply and limited weight-bearing activity, which typically lead to a transient decrease in BMD during the first three to six months [14]. Nonetheless, other reports suggest that bone remodeling may continue beyond the first year, with BMD gradually stabilizing over a longer period [14,27].

Long-term follow-up data on periprosthetic BMD remain limited. One notable study by Kim et al. compared short and standard stems over 17 years and reported BMD decreases in ROIs 1 and 7, although the reduction reached statistical significance only in the standard-stem group [9]. Importantly, the short stem in their study featured metaphyseal anchorage, which differs from the calcar-guided design used in our series. Koyano et al., who used a type-3 stem, found evidence of stress shielding in all femurs at 9.2 years, but they also observed proximal cancellous bone condensation with the anatomic stem, suggesting a more proximal load transfer [3]. On the other hand, the Microplasty stem (type-3) evaluated by Hirao et al. showed comparable BMD changes from baseline in both groups at five years [19].

Regarding functional outcomes, both implant types achieved comparable and satisfactory HHS, with no significant intergroup differences. This aligns with previous studies and is further supported by the comparable incidence of postoperative thigh pain observed in both groups [20,27]. Nevertheless, the overall rate of thigh pain in our study was higher than typically reported in the literature. For instance, Kim et al. measured a thigh pain rate ranging from 0.7% to 15% [9]. This discrepancy may be attributable to our small sample size, other causes of the pain, patient factors, activity level, or implant design (other than length).

A key strength of this study is its long-term follow-up, which provides meaningful insight into bone remodeling. The inclusion of patients without an upper age limit contributes to the sample's heterogeneity, making it more representative of the general population undergoing THA. Additionally, we adapted the conventional Gruen zones to what we believe is a more accurate evaluation of BMD across different femoral morphologies, regardless of implant type. Indeed, traditional Gruen zone analysis can introduce biases due to natural variations in BMD between the proximal and distal femur. Nonetheless, this study has several limitations. Its retrospective, single-center design limited patient recruitment, resulting in a relatively small final sample size of individuals who had undergone bilateral THA with both the TwinSys and Optimys stems for the pilot study. Despite efforts to broaden the inclusion criteria, the resulting small sample size may limit the generalizability of the findings, especially since the group is not entirely uniform (in sex distribution, follow-up duration, and possibly cup type and patient factors). Additionally, baseline BMD values were not available in patient records, preventing assessment of longitudinal changes. Moreover, DEXA scans were restricted to the anteroposterior view, which may not fully capture three-dimensional changes in bone structure. Besides, the study focused on a single short-stem design. While this reflects local clinical practice, it limits the broader applicability of the results. Finally, the only design variable considered was stem length; however, other factors, such as stem geometry and surface coating, may also influence bone remodeling, and some patient factors may also have an influence (smoking, osteoporosis, vitamin D status, antiresorptive medication, inflammatory disease, baseline activity level, systemic bone health) but were not addressed in this study.

## Conclusions

The Optimys short stem showed comparable long-term clinical and radiographic findings, with no clear BMD superiority over the TwinSys standard stem at a mean follow-up of 9.4 years. This supports the durable performance of this specific short-stem design, but does not prove superior proximal bone preservation. However, this comparison cannot be generalized to all stem designs. Registry data indicate that the survivorship of uncemented implants often exceeds the current follow-up period of our study. Continued monitoring of periprosthetic bone remodeling over time is therefore essential.

## References

[1] Ehlinger M, Delaunay C, Karoubi M, Bonnomet F, Ramdane N, Hamadouche M (2014). Revision of primary total hip arthroplasty for peri-prosthetic fracture: a prospective epidemiological study of 249 consecutive cases in France. Orthop Traumatol Surg Res.

[2] Joshi MG, Advani SG, Miller F, Santare MH (2000). Analysis of a femoral hip prosthesis designed to reduce stress shielding. J Biomech.

[3] Koyano G, Jinno T, Koga D, Yamauchi Y, Muneta T, Okawa A (2017). Comparison of bone remodeling between an anatomic short stem and a straight stem in 1-stage bilateral total hip arthroplasty. J Arthroplasty.

[4] Meyrueis P, Cazenave A, Zimmermann R (2004). Biomechanics of bones and treatment of fractures. EMC - Rhumatologie-Orthopedie.

[5] Liang HD, Yang WY, Pan JK, Huang HT, Luo MH, Zeng LF, Liu J (2018). Are short-stem prostheses superior to conventional stem prostheses in primary total hip arthroplasty? A systematic review and meta-analysis of randomised controlled trials. BMJ Open.

[6] Engh CA, Bobyn JD, Glassman AH (1987). Porous-coated hip replacement. The factors governing bone ingrowth, stress shielding, and clinical results. J Bone Joint Surg Br.

[7] Knutsen AR, Lau N, Longjohn DB, Ebramzadeh E, Sangiorgio SN (2017). Periprosthetic femoral bone loss in total hip arthroplasty: systematic analysis of the effect of stem design. Hip Int.

[8] Senesi G, Barone G, Pinelli S (2025). The relationship between stress shielding, bone density changes and implant migration, failure and fracture after total knee arthroplasty: a systematic review. J Exp Orthop.

[9] Kim YH, Jang YS, Kim EJ (2021). A prospective, randomized comparison of the long-term clinical and radiographic results of an ultra-short vs a conventional length cementless anatomic femoral stem. J Arthroplasty.

[10] Erivan R, Villatte G, Dartus J (2022). French Hip and Knee Society classification of short-stem hip prostheses: inter- and intra-observer reproducibility. Orthop Traumatol Surg Res.

[11] Stulberg SD, Patel RM (2013). The short stem: promises and pitfalls. Bone Joint J.

[12] McTighe T, Stulberg SD, Keppler L (2013). A classification system for short stem uncemented total hip arthroplasty. Orthop Proc.

[13] Falez F, Casella F, Papalia M (2015). Current concepts, classification, and results in short stem hip arthroplasty. Orthopedics.

[14] Lerch M, Haar-Tran A, Windhagen H, Behrens BA, Wefstaedt P, Stukenborg-Colsman CM (2012). Bone remodelling around the Metha short stem in total hip arthroplasty: a prospective dual-energy X-ray absorptiometry study. Int Orthop.

[15] Zeh A, Pankow F, Röllinhoff M, Delank S, Wohlrab D (2013). A prospective dual-energy X-ray absorptiometry study of bone remodeling after implantation of the Nanos short-stemmed prosthesis. Acta Orthop Belg.

[16] Salemyr M, Muren O, Ahl T, Bodén H, Eisler T, Stark A, Sköldenberg O (2015). Lower periprosthetic bone loss and good fixation of an ultra-short stem compared to a conventional stem in uncemented total hip arthroplasty. Acta Orthop.

[17] Kim SS, Kim HJ, Kim KW, Jung YH, Heo SY (2020). Comparative analysis between short stem and conventional femoral stem in patients with osteonecrosis of femoral head: Metha stem and Excia stem. Orthop Surg.

[18] Lacko M, Filip V, Gharaibeh A, Lackova A, Folvarsky M, Zamborsky R (2021). Comparison of bone remodelling around short stem and conventional straight stem in total hip replacement: a prospective randomized radiographic and dual-energy X-ray absorptiometric study. Bratisl Lek Listy.

[19] Hirao M, Miyatake K, Koga D, Takada R, Koyano G, Okawa A, Jinno T (2021). Comparison of 5-year postoperative results between standard-length stems and short stems in one-stage bilateral total hip arthroplasty: a randomized controlled trial. Eur J Orthop Surg Traumatol.

[20] Freitag T, Hein MA, Wernerus D, Reichel H, Bieger R (2016). Bone remodelling after femoral short stem implantation in total hip arthroplasty: 1-year results from a randomized DEXA study. Arch Orthop Trauma Surg.

[21] Kim YH, Choi Y, Kim JS (2011). Comparison of bone mineral density changes around short, metaphyseal-fitting, and conventional cementless anatomical femoral components. J Arthroplasty.

[22] Kim YH, Park JW, Kim JS (2016). Ultrashort versus conventional anatomic cementless femoral stems in the same patients younger than 55 years. Clin Orthop Relat Res.

[23] Dorr LD, Faugere MC, Mackel AM, Gruen TA, Bognar B, Malluche HH (1993). Structural and cellular assessment of bone quality of proximal femur. Bone.

[24] Erivan R, Villatte G, Dartus J, Reina N, Descamps S, Boisgard S (2019). Progression and projection for hip surgery in France, 2008-2070: epidemiologic study with trend and projection analysis. Orthop Traumatol Surg Res.

[25] Batailler C, Shatrov J, Schmidt A, Servien E, Puch JM, Lustig S (2021). Similar stress repartition for a standard uncemented collared femoral stem versus a shortened collared femoral stem. SICOT J.

[26] Chen HH, Morrey BF, An KN, Luo ZP (2009). Bone remodeling characteristics of a short-stemmed total hip replacement. J Arthroplasty.

[27] Samy AM, El-Tantawy A (2019). Stem length in primary cementless total hip arthroplasty: does it make a difference in bone remodeling?. Eur J Orthop Surg Traumatol.

[28] Jahnke A, Engl S, Altmeyer C, Jakubowitz E, Seeger JB, Rickert M, Ishaque BA (2014). Changes of periprosthetic bone density after a cementless short hip stem: a clinical and radiological analysis. Int Orthop.

[29] Djebara AE, El Yagoubi A, Mertl P, El Fatayri B, Dehl M, Gabrion A (2022). Comparison of periprosthetic bone mineral density between two types of short-stems in total hip arthroplasty with a mean follow-up of 4 years. Orthop Traumatol Surg Res.

[30] Kim YH, Kim JS, Joo JH, Park JW (2012). A prospective short-term outcome study of a short metaphyseal fitting total hip arthroplasty. J Arthroplasty.

